# Optimal time of tumour response evaluation and effectiveness of hypofractionated proton beam therapy for inoperable or recurrent hepatocellular carcinoma

**DOI:** 10.18632/oncotarget.23428

**Published:** 2017-12-19

**Authors:** Tae Hyun Kim, Joong-Won Park, Bo Hyun Kim, Dae Yong Kim, Sung Ho Moon, Sang Soo Kim, Ju Hee Lee, Sang Myung Woo, Young-Hwan Koh, Woo Jin Lee, Chang-Min Kim

**Affiliations:** ^1^ Center for Liver Cancer, Research Institute and Hospital, National Cancer Center, Goyang, Korea

**Keywords:** hepatocellular carcinoma, tumour response, proton beam therapy

## Abstract

**Objective:**

To evaluate the optimal time of tumour response and effectiveness of hypofractionated proton beam therapy (PBT) for hepatocellular carcinoma (HCC).

**Results:**

Overall, treatment was well tolerated with no grade toxicity ≥3. Of 71 patients, 66 patients (93%) eventually reached complete response (CR) after PBT: 93.9% (62 of 66) of patients who reached CR within 12 months, and the remaining 4 patients (6.1%) reached CR at 12.5, 16.2, 19.1 and 21.7 months, respectively. The three-year local progression-free survival (LPFS), relapse-free survival (RFS) and OS rates were 89.9%, 26.8%, and 74.4%, respectively. Multivariate analysis revealed that the tumour response was an independent prognostic factor for LPFS, RFS, and OS.

**Conclusion:**

Most CR was achieved within 1 year after PBT and further salvage treatments in PBT field might be postponed up to approximately 18–24 months. Hypofractionated PBT could be good alternative for HCC patients who are unsuitable for surgical or invasive treatments with curative intent.

**Materials and Methods:**

Seventy-one inoperable or recurrent HCC patients underwent hypofractionated PBT using 66 GyE in 10 fractions. The tumour responses were defined as the maximal tumour response observed during the follow-up period using the modified Response Evaluation Criteria in Solid Tumors criteria.

## INTRODUCTION

Surgical resection, liver transplantation, and local ablative treatments, including radiofrequency ablation (RFA), are considered as curative treatment options for patients with hepatocellular carcinoma (HCC) [1]. Unfortunately, the use of surgical resection and liver transplantation are restricted to selected patients due to the multifocality of HCC development in cirrhotic livers, advanced tumours and/or comorbidities, including underlying liver cirrhosis (LC), and the shortage of graft donors. In addition, RFA is unsuitable for patients with bleeding tendency, unfavourable tumour location (i.e., proximity of lesions to the major vessels or gall bladder, and sub-diaphragmatic lesions), presence of a non-echogenic lesion, or large-sized tumours. For inoperable HCC patients unsuitable for local ablative therapies, although transcatheter arterial chemoembolisation (TACE) has a survival benefit compared with best supportive care [2, 3], its radical effects are limited histopathologically [4]. Thus, there seems to be a need for effective and less invasive local treatments in inoperable HCC patients ineligible for local ablative therapies.

Technological innovations in the field of radiotherapy (RT), such as three-dimensional conformal RT (CRT), intensity-modulated radiation therapy (IMRT), stereotactic body radiation therapy (SBRT), and proton beam therapy (PBT), could potentially deliver radiation more precisely to tumours while sparing the normal tissues [5–17]. Recently, hypofractionated CRT/IMRT with ∼10 fractions or SBRT with 3–6 fractions have been attempted to reduce the treatment duration to be more convenient for patients and to increase the biologic effect of RT [9, 12, 14, 18–20]. Moreover, due to the distinct physical characteristics of proton beams and the Bragg peak in allowing deposition of high doses of radiation within the target and the lack of an exit dose outside the target, the role of charged particle therapy, including PBT, has been actively investigated [5–7, 13, 15, 16]. Conceptually, hypofractionated PBT can create a potential improvement in the therapeutic ratio compared with conventional fractionated PBT due to less repair of radiation damage of surrounding normal tissues and shortened overall treatment time. Based on this background, inoperable or recurrent HCC patients who failed or were unsuitable to local therapies have been treated at our institution by hypofractionated PBT. In addition, although several studies have reported that objective response gradually increased up to 1 year after SBRT and PBT [5, 9, 21], to the best of our knowledge, the optimal time of tumour response evaluation after RT, including PBT, and impact of time interval between tumour response and RT has not been thoroughly evaluated by a long-term follow-up study. This information is important when deciding further salvage treatments after RT. This study was designed to retrospectively analyse the clinical outcomes of hypofractionated PBT in inoperable or recurrent HCC patients and to evaluate the optimal time of tumour response evaluation and clinical effectiveness of this method.

## RESULTS

Patient characteristics are summarised in Table 1. The study included 60 men (84.5%) and 11 women (15.5%) with a median age of 63 years (range, 40–92 years). Sixty-eight patients were in Child-Pugh class A, and 3 patients were Child-Pugh class B. The AFP level ranged from 1.3 to 2732.2 ng/mL (median, 7.6 ng/mL). All but 1 patient, who was treatment naïve due to refusal to surgical and nonsurgical treatments, had recurrent or residual tumour in the PBT site. Of the 71 patients, 55 (77.5%) had a recurrent or residual tumour in the liver prior to PBT despite undergoing one or more interventions to PBT site, including TACE, RFA, and/or PEIT. The remaining 16 patients (15.5%) had not received any prior treatment to the PBT site due to the lack of indications for other treatment modalities (Table 1). After completion of PBT, additional treatments were not performed at the PBT site until tumour progression was confirmed.

**Table 1:** Patient characteristics

Characteristics		Total, *n* (%)
Gender	Male	60 (84.5)
	Female	11 (15.5)
Age, years	Median (range)	63 (40–92)
	<60	22 (31.0)
	≥60	49 (69.0)
ECOG PS	0	71 (100)
Aetiology of LC	HBV	57 (80.3)
	HCV	5 (7.0)
	Alcoholic	3 (4.2)
	unknown	6 (8.5)
Child–Pugh Classification	A	68 (95.8)
	B	3 (4.2)
AFP, ng/mL	Median (range)	7.6 (1.3–2732.2)
	<10	43 (60.6)
	≥10	28 (39.4)
Tumour size, cm	Median (range)	1.5 (1.0–8.5)
	<3	64 (90.1)
	≥3	7 (9.9)
AJCC stage	I	15 (21.1)
	II	51 (71.8)
	IIIA	5 (7.1)
BCLC stage	A	43 (60.6)
	B	28 (39.4)
Pre-Tx to PBT site	No	16 (22.5)
	Yes	55 (77.5)
	TACE	49 (89.1)
	TACE + RFA and/or PEIT	6 (10.9)
Pre-Tx to other site	No	11 (15.5)
	Yes	60 (84.5)
	TACE ± RFA ± PEIT	44 (73.3)
	SR ± TACE ± RFA ± PEIT ± Sorafenib	14 (23.3)
	RFA	2 (3.4)

Abbreviations: LC, liver cirrhosis; HBV, hepatitis B virus; HCV, hepatitis C virus; AFP, a-fetoprotein; ECOG, Eastern Cooperative Oncology Group; PS, performance status; AJCC, American Joint Committee on Cancer; BCLC, Barcelona Clinic Liver Cancer; Tx, treatment; PBT, proton beam therapy; TACE, transcatheter arterial chemoembolisation; RFA, radiofrequency ablation; PEIT; percutaneous ethanol injection treatment; and SR, surgical resection.

**Figure 1:**
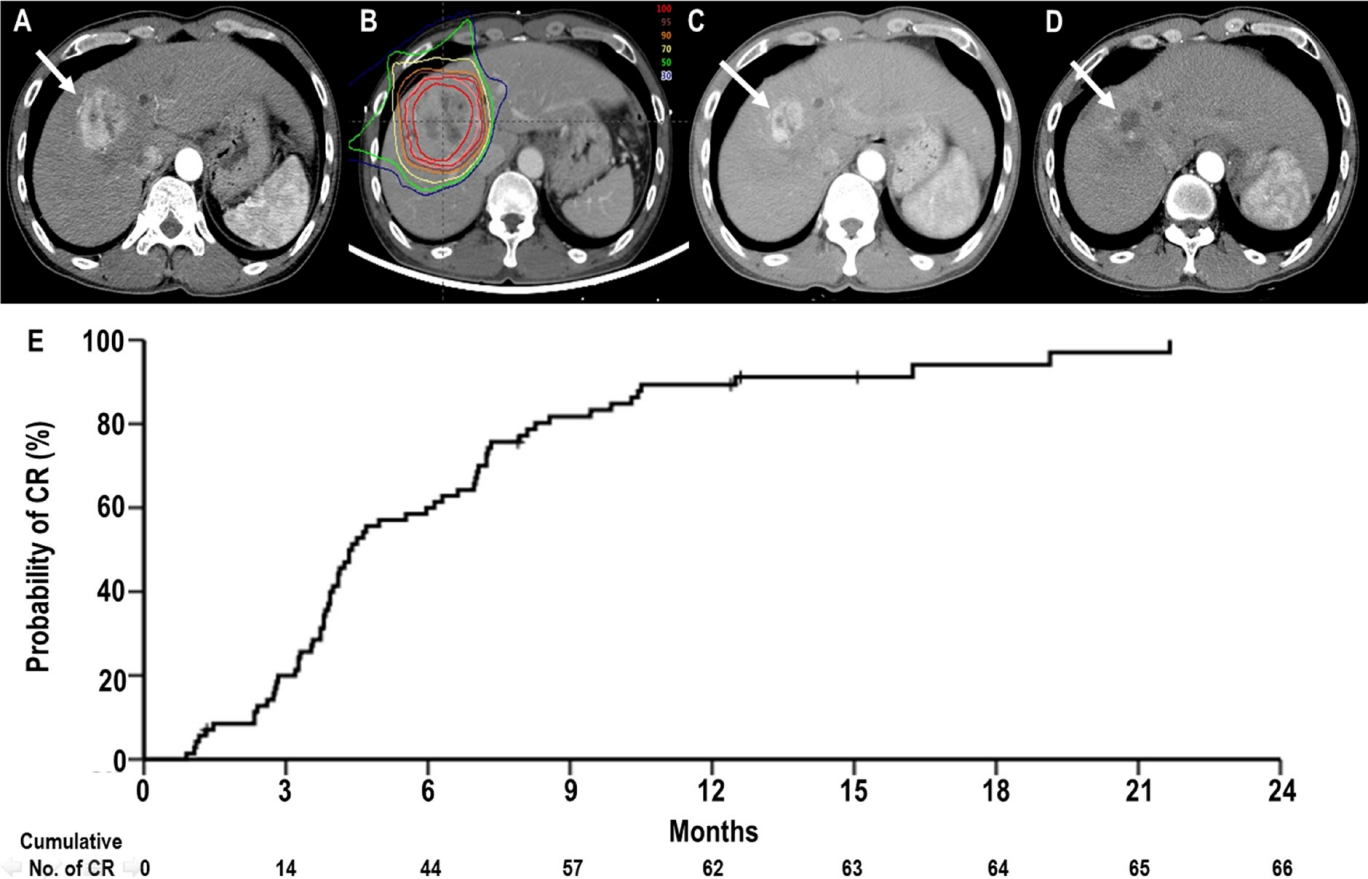
Complete response (CR) of a primary tumour to proton beam therapy (PBT) (**A**) Pretreatment CT scans showing the primary tumour (arrow). (**B**) The patient underwent PBT. (**C**) CT scans at 3 months after PBT demonstrating shrinkage of the primary tumour (arrow). (**D**) CT scans at 8 months after PBT demonstrating complete regression of the primary tumour (arrow). (**E**) The actuarial CR curves of primary tumour after PBT.

The median follow-up duration was 31.3 months (range, 4.2–47 months). Of 71 patients, 66 patients (93%) reached CR eventually (Figure 1). The remaining 5 patients (7%) did not reach CR: SD in 1 (1.4%) and PD in 4 (5.6%). Of the 66 patients who reached CR, the mean time to CR was 6.3 months (95% confidence interval [CI], 5.2–7.5 months) (range, 1–21.7 months): 18 patients (27.3%) showed CR within 3 months, 44 patients (66.7%) showed CR within 6 months, 57 patients (86.4%) showed CR within 9 months, 62 patients (93.9%) showed CR within 12 months, and remaining 4 patients (6.1%) reached CR at 12.5, 16.2, 19.1 and 21.7 months after PBT, respectively. The actuarial CR rates at 3 months, 6 months, 9 months and 12 months were 21.3% (95% CI, 11.7–30.9%), 60% (95% CI, 48.4–71.6%), 81.8% (95% CI, 72.6–91.0%) and 89.4% (95% CI, 81.9–96.8%), respectively (Figure 1). The distributions of clinical characteristics were not significantly different between the patients who reached CR and those who did not reach CR (*p* > 0.05 each) (Supplementary Table 1).

**Figure 2:**
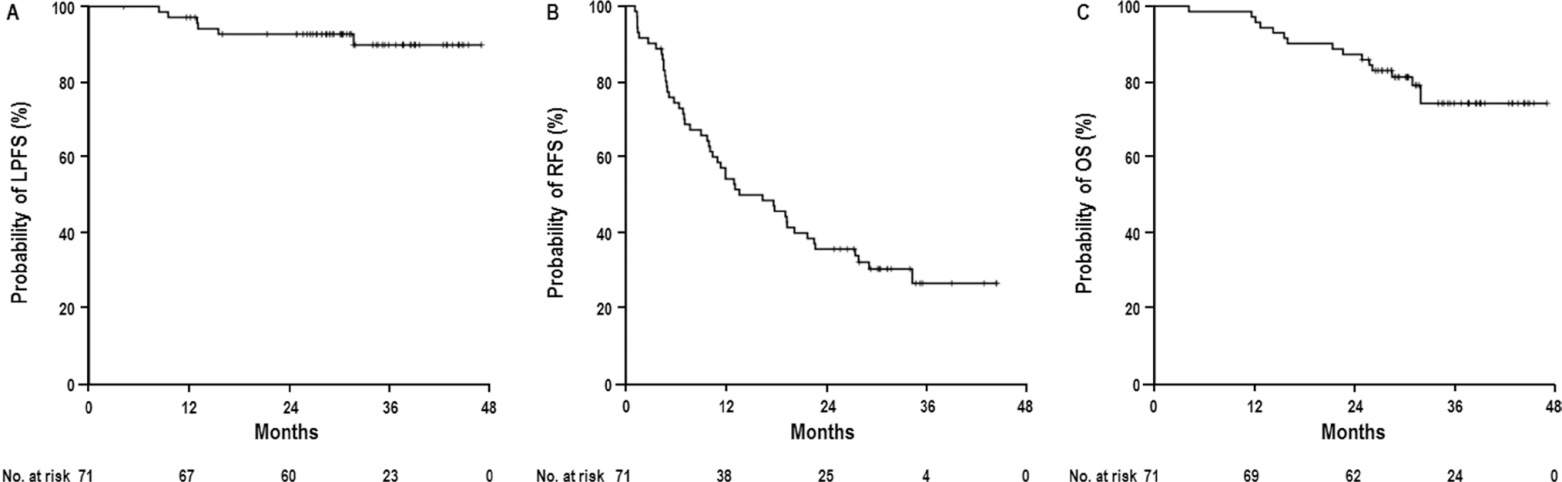
Local progression-free survival (LPFS) (**A**), relapse-free survival (RFS) (**B**), and overall survival (OS) (**C**) curves in all patients.

At the time of analysis, 16 patients died due to intrahepatic disease progression (*n* = 10), liver failure by progression of LC (*n* = 2), bone metastasis (*n* = 2), brain metastasis (*n* = 1), and pneumonia (*n* = 1), not related with treatment, and 55 remained alive. Of the 71 patients, 49 (69%) developed disease recurrence, including 6 (8.5%) with local progression, 49 (69%) with intrahepatic recurrence, and 11 (15.5%) with distant metastases (Supplementary Figure 1). The median time to local progression, intrahepatic progression, and distant metastasis was 13 months (range, 8.4–31.7 months), 9.9 months (range, 1.1–34.3 months), and 12.1 months (range, 7.7–32.5 months), respectively. After the diagnosis of disease recurrences, 47 of 49 (95.9%) patients, except for 2 patients (combined with obstructive jaundice by progressive disease [*n* = 1], or progressive distant metastasis [*n* = 1]), received salvage treatments to the PBT site and/or other sites, such as one or combinations of various locoregional treatments (i.e., TACE, RFA, PBT) and/or systemic treatments (i.e., sorafenib, doxorubicin, etc.). The actuarial 3-year LPFS, RFS and OS rates were 89.9% (95% CI, 81.8–98%), 26.8% (95% CI, 14.9–38.7%), and 74.4% (95% CI, 63.1–85.7%), respectively (Figure 2). Local progression developed in 2 of 66 patients (3%) who reached CR and 4 of 5 patients (80%) who did not reach CR (*p* < 0.001). Of 66 patients who reached CR, there was no significant differences in 3-year LPFS (97.6% [95% CI, 93.0–100%] vs. 93.3% [95% CI, 80.7–100%], *p* = 0.793), DFS (24.3% [95% CI, 6.8–41.8%] vs. 32.1% [95% CI, 12.9–51.4%], *p* = 0.336), OS (80.4% [95% CI, 68.1–92.6%] vs. 81.2% [95% CI, 64.5–97.9%], *p* = 0.416) rates between the patients who reached CR within 6 months after completion of RT and those who reached CR after 6 months. Before CR was reached in PBT site, intrahepatic progression was observed in 11 of 66 patients (16.7%) who reached CR, was treated with local treatments, such as TACE (*n* = 8), RFA (*n* = 2), and combination of TACE and RFA (*n* = 1), and distant metastasis was not observed. Of 66 patients who reached CR, there was no significant difference in 3-year LPFS rates (100% vs. 94.8%, *p* = 0.338) whether salvage treatments to other sites was given or not. Three-year LPFS (95.5% vs. 0%, *p* < 0.001), RFS (28.3% % vs. 0%, *p* = 0.046), and OS (81.1% vs. 0%, *p* < 0.001) rates were significantly increased in the patients who reached CR compared with those who did not. However, the 3-year LPFS, RFS, and OS rates were not significantly different whether pre-treatments to the PBT site or other sites were given or not (Table 2).

**Table 2:** Univariate analysis of clinical characteristics associated with local progression-free survival (LPFS), relapse-free survival (RFS), and overall survival (OS)

			LPFS		RFS		OS	
Characteristics		No. of patients, *n*	3 year (95% CI), %	*p*-value^†^	3 year (95% CI), %	*p*-value^†^	3 year (95% CI), %	*p*-value^†^
Gender	Male	60	87.8 (78.1–97.5)	0.273	21.9 (6.6–37.2)	0.896	72.6 (59.7–85.5)	0.694
	Female	11	100 (100)		36.4 (8.0–64.8)		81.8 (59.0–100)	
Age, years	<60	22	89.5 (79.2–99.8)	0.879	21.8 (4.1–39.5)	0.387	81.8 (65.7–97.9)	0.589
	≥60	49	90.7 (78.4–100)		28.8 (13.4–44.2)		71.3 (60.0–85.6)	
Aetiology of LC	HBV	57	87.3 (77.2–97.4)	0.214	24.3 (11.6–37.0)	0.497	71.4 (58.2–84.7)	0.445
	Others	14	100 (100)		38.7 (12.2–65.2)		85.7 (67.4–100)	
Child–Pugh Classification	A	68	89.6 (81.3–97.9)	0.665	27.5 (15.3–39.7)	0.051	76.4 (65.1–87.7)	0.034
	B	3	100 (100)		0 (–)		33.3 (0–86.6)	
AFP, ng/mL	<10	43	88.8 (77.9–99.7)	0.882	33.7 (19.6–48.2)	0.098	84.2 (72.2–96.2)	0.032
	≥10	28	92.1 (82.1–100)		17.0 (0–34.6)		60.0 (39.8–80.2)	
Tumour size, cm	<3	64	88.8 (80.0 -97.7)	0.426	24.6 (11.6–37.6)	0.413	74.6 (62.6–86.6)	0.697
	≥3	7	100 (100)		42.9 (6.4–79.6)		71.4 (38.0–100)	
AJCC stage	I–II	66	89.3 (80.8–97.8)	0.534	25.9 (13.2–38.4)	0.658	77.8 (66.7–88.9)	0.030
	IIIA	5	100 (100)		30.0 (0–76.8)		30.0 (0–76.8)	
BCLC stage	A	43	91.3 (81.5–100)	0.447	24.0 (8.8–39.3)	0.899	82.0 (69.6–94.4)	0.090
	B	28	88.6 (76.4–100)		29.9 (10.9–48.8)		61.6 (40.3–82.9)	
Pre-Tx to PBT site	No	16	88.9 (68.4–100)	0.647	31.3 (8.6–54.0)	0.817	93.8 (81.9–100)	0.087
	Yes	55	90.4 (82.4–98.4)		25.7 (11.7–39.6)		68.7 (91.9–82.5)	
Pre-Tx to other site	No	11	90.9 (73.9–100)	0.930	54.6 (25.2–84.0)	0.073	81.8 (59.0–100)	0.749
	Yes	60	89.8 (80.9–98.7)		22.5 (10.5–34.5)		73.5 (61.2–85.8)	
Tumour response	CR	66	95.5 (89.1–100)	<0.001	28.3 (15.8–40.9)	0.046	81.1% (70.6–91.5)	<0.001
	Non-CR	5	0 (–)		0 (–)		0 (–)	

Abbreviations: CI, confidence interval; CR, complete response; all others are the same as in Table 1.

^†^log-rank test.

Univariate and multivariate analyses were performed to identify clinical parameters predicting LPFS, RFS, and OS (Tables 2 and 3, respectively). Univariate analysis demonstrated that tumour response was significantly associated with LPFS and RFS and that Child-Pugh classification, AFP level, AJCC stage, and tumour response were significantly associated with OS (*p* < 0.05) (Table 2). In multivariate analysis, tumour response was a significant factor independently associated with LPFS and RFS (*p* < 0.05 each). In addition, Child-Pugh classification, AJCC stage and tumour response were significant factors independently associated with OS (*p* < 0.05 each) (Table 3).

**Table 3:** Multivariate analysis of clinical characteristics associated with local progression-free survival (LPFS), relapse-free survival (RFS), and overall survival (OS)

Characteristics		LPFS		RFS		OS	
		Hazard Ratio (95% CI)	*p*-value^†^	Hazard Ratio (95% CI)	*p*-value^†^	Hazard Ratio (95% CI)	*p*-value^†^
Child-Pugh Classification	A	-	-	-	-	1.000	0.017
	B	-		-		6.992 (1.423–34.340)	
AJCC stage	I–II	-	-	-	-	1.000	0.005
	IIIA	-		-		7.059 (1.788–27.870)	
Tumour response	CR	1.000	<0.001	1.000	0.027	1.000	<0.001
	Non-CR	115.149 (12.300–1077.953)		3.392 (1.148–10.026)		15.801 (4.886–51.103)	

Abbreviations: same as in Tables 1 and 2.

^†^Cox proportional hazards model.

Overall, treatment was well tolerated with no patient experiencing grade ≥3 toxicity. Within 3 months after PBT, acute toxicities were transient, easily manageable, and caused no interruption in treatment course. Of the 71 patients, 62 (87.3%) showed no change in Child–Pugh score, six (8.5%) showed a 1-point decrease and three (4.2%) showed a 1-point increase. Three (4.2%) patients experienced grade 1 elevated ALT without evidence of tumour progression, and six (8.5%) patients experienced grade 1 leukopenia and thrombocytopenia. After 3 months after PBT, no late gastrointestinal toxicities defined as gastric or duodenal ulcers within the RT field, late hepatic failure induced by radiation-induced liver disease or treatment-related death was observed.

## DISCUSSION

The optimal time of response evaluation after RT for HCC has not been well-defined. Price *et al.* [21] analysed 26 HCC patients treated with SBRT using 24–48 Gy in 3–5 fractions and reported that the objective response (CR + PR) rates at 3 months, 6 months, 9 months, and 12 months were 59 ± 8%, 69 ± 8%, 81 ± 8%, and 92 ± 7%, respectively. Similarly, Kwon *et al.* [9] analysed 42 HCC patients treated with SBRT using 30–39 Gy in 3 fractions and reported that the mean time to achieve objective response was 5.1 ± 3.7 months. Kawashima *et al.* [5] analysed 30 patients treated with PBT using 76 GyE in 20 fractions and also demonstrated that 24 (80%) patients achieved CR at 5–20 months (median, 8 months) after PBT. The present study analysed 71 HCC patients treated with PBT using 66 GyE in 10 fractions. The actuarial CR rates at 3 months, 6 months, 9 months, 12 months were 21.3% (95% CI, 11.7–30.9%), 60% (95% CI, 48.4–71.6%), 81.8% (95% CI, 72.6–91.0%) and 89.4% (95% CI, 81.9–96.8%), respectively. The mean time to CR was 6.3 months (95% CI, 5.2–7.5 months) (Figure 1). Of 66 patients who reached CR, 62 (93.9%) showed CR within 12 months and remaining 4 patients (6.1%) reached CR at 12.5, 16.2, 19.1 and 21.7 months after PBT, respectively. The patients who reached CR had significantly higher 3-year LPFS rates compared with patients who did not reach CR (95.5% vs. 0%, *p* < 0.001). Considering these findings, evaluation of response might be performed every 3 months for the first year after completion of PBT. In cases which do not achieve a CR within 1 year, further salvage treatment could be postponed up to approximately 18–24 months after completion of PBT.

For HCC patients with a single tumour and well-preserved liver function, surgical resection result in 5-year survival rates of 50–70% [1, 22–24]. As an alternative to surgical treatment, RFA has demonstrated satisfactory local tumour control of 70–90% and a similar 5-year survival rate of 50–75% compared with surgical resection in patients with small HCC(s) <3 cm away from large vessels and Child Pugh class A [1, 24–26]. However, although RFA results in first CR in approximately 80% of HCCs ≤3 cm in diameter, local progression was noted in 30–40% of patients with first CR experience, approximately 70% of patients with HCCs of 3.1–5 cm in diameter and all patients with HCCs >5 cm [27, 28]. Recently, SBRT with 24–60 Gy in 3–6 fractions has been tried as an emerging non-invasive alternative to RFA and has demonstrated a similar 2- or 3-year LPFS of 58–92% and 2- or 3-year OS of 53–70% compared with RFA [9, 12, 14, 18–21]. Whal *et al.* reported no difference in 2-year LPFS (80.2% vs. 83.8%, *p* > 0.05) and OS (52.9% vs. 46.3%, *p* > 0.05) between patients treated with RFA and those with SBRT [11]. Similar to SBRT, PBT with 52.8–84 GyE in 4–34 fractions has been attempted for inoperable or recurrent HCC patients and has demonstrated a promising 2- or 3-year LPFS of 75–96% and 45.1–66% [5, 7, 13, 15, 16, 29]. In the present study, we applied hypofractionated PBT with 66 GyE in 10 fractions and observed a 3-year LPFS rate of 89.9% and 3-year OS rate of 74.4%. Although direct comparison of data among previous studies is difficult due to heterogeneous baseline characteristics, particularly regarding the degree of liver function impairment and tumour burden, in the present study, the LPFS and OS were comparable with outcomes of curative treatments, such as surgical resection and RFA, for HCC patients in our institutional cohort data [24] and other studies [1, 3, 11, 26, 27, 30]. In addition, multivariate analysis revealed that tumour response after PBT was a significant factor independently associated with LPFS, RFS, and OS (*p* < 0.05 each), suggesting that PBT could improve LPFS and subsequently RFS and OS. These findings suggest that PBT may have a major role in treatment of both favourable (small, solitary tumours and good liver functions) and difficult-to-treat cases of HCC, such as those with large or recurrent tumour(s) after additional curative treatments.

Similar to previous studies [5, 7, 9, 13, 14, 18, 20, 21, 29], the major pattern of failure in the present study was intrahepatic recurrence outside the RT field (69%) given the multifocal nature of HCC in the cirrhotic liver and high proportion (84.5%) of intrahepatic disease outside of RT field in our study population. To enable further subsequent salvage treatment for residual or recurrent tumours after RT, such as TACE, sorafenib, RFA, surgery, and RT, it should be necessary to spare normal liver tissues to maintain remnant liver function. In a recent meta-analysis [16], survival rates for charged particle therapy were increased compared with those for CRT but similar to those for SBRT, and toxicity tends to be lower for charged particle therapy compared with CRT and SBRT. Similarly, our previous study demonstrated that better dose localisation properties of protons compared with photons made it possible to more effectively spare normal liver tissue in PBT compared with RT with photons [31]. Although the present study included several unfavourable prognostic characteristics (e.g., recurrent tumours and advanced stage), 47 of 49 (95.9%) patients who had recurrent disease received post-PBT treatment to the PBT site and/or other sites; moreover, treatment-related toxicities were minimal, with no grade ≥3 toxicity. These findings suggested that PBT could achieve local tumour control safely without influencing further salvage treatments for intrahepatic recurrence. However, because our data were obtained from a single institutional retrospective study with a relatively small population including small subgroup of Child-Pugh class B (*n* = 3), the effects of local and systemic treatment for intrahepatic and/or metastatic disease, impact of remnant liver function, such as Child-Pugh Class B, and probable selection bias were not thoroughly evaluated. Thus, we initiated a phase III study to confirm the effectiveness and feasibility of PBT for HCC patients with recurrent or residual disease compared with RFA (NCCTS-13-695).

In conclusion, most (94%) patients achieved CR within 1 year after hypofractionated PBT, with median time to CR of about 6 months, and remaining 6.1% patients achieved CR at 12–22 months. There were no significant differences in LPFS, DFS and OS between the patients who reached CR within 6 months after completion of RT and those who reached CR after 6 months. These findings suggested that further salvage treatments in PBT field might be postponed up to approximately 18–24 months. Hypofractionated PBT exhibited feasible and promising outcomes in terms of LPFS and OS for inoperable or recurrent HCC, which were comparable with outcomes of curative treatments, such as surgical resection or RFA, for HCC patients with early stage and well-preserved liver function. However, to accurately assess the optimal time of tumour response evaluation and effectiveness of hypofractionated PBT on LPFS, RFS, and OS compared with other local modalities, further larger and comprehensive studies are needed. However, our data suggested that hypofractionated PBT could be good alternative modality for HCC patients who are unsuitable for curative treatments, such as surgical resection or local ablative therapy.

## MATERIALS AND METHODS

### Patients

Between May 2013 and February 2015, a total of 71 patients with HCC receiving hypofractionated PBT who met the following criteria were included in this study: HCC was diagnosed by pathologic confirmation (*n* = 17) or on the basis of radiologic findings plus serum alpha-fetoprotein (AFP) concentrations ≥200 ng/mL (*n* = 54) in accordance with the guidelines of the Korean Liver Cancer Study Group and the National Cancer Center [23]; gross tumour ≥2 cm from gastrointestinal structures; liver function of Child-Pugh class A or B; no active tumours outside the target volume; no history of previous RT to the target volume; no extrahepatic metastases; and no uncontrolled ascites. HCCs were classified according to the 2010 American Joint Committee on Cancer (AJCC) and Barcelona Clinic Liver Cancer (BCLC) [1] staging system and liver function was classified according to Child–Pugh classification. The study was performed in accordance with the guidelines of our institutional review board, which waived the requirement for informed consent due to the retrospective nature of the study.

### Pretreatment evaluation and treatment

All patients underwent blood tests, including measurements of blood cell counts, liver and renal function tests, titres of hepatitis B and C virus (HBV and HCV, respectively), and AFP. Abdominal dynamic contrast-enhanced CT and/or MRI was used to evaluate the extent of HCC. For RT planning, patients underwent CT simulation in a supine position with arms above the head and immobilised using an arm-up holder to improve setup reproducibility. Contrast-enhanced four-dimensional (4D) CT images were acquired with 2.5-mm slice thickness under shallow respiration using a 4D CT simulator (Light-Speed RT; GE Healthcare, Waukesha, WI, USA). During the 4D CT scan, the respiration signals of the patients were monitored by a real-time position management (RPM) system (Varian Medical Systems, Palo Alto, CA, USA). The acquired CT images were reconstructed in 10 equally spaced respiratory phases and, in the post-processing stage, maximum intensity projection (MIP), minimum intensity projection (MinIP), and average intensity projection (AIP) CT images were reconstructed using exhalation (gated) phases (30% of total respiratory cycle) on an Advantage workstation (Version 4.3, GE Healthcare, Milwaukee, WI, USA). All 4D CT images were transferred to the Eclipse treatment planning system (Version 8.1; Varian Medical System, Palo Alto, CA, USA), and the contours for targets and organs at risk (OARs) were delineated in AIP-CT images during the exhalation (gated) phases. Figure 1 illustrates the definition of target volumes. The gross tumour volume (GTV) included all detectable primary tumours as determined by contrast-enhanced AIP-CT images during the exhalation phase, and the clinical target volume (CTV) was regarded as GTV [6–8, 10, 31, 32]. The gated internal target volume (ITV) was obtained by summing the GTVs in each CT image during the exhalation phases. The planning target volume included the ITV plus 5–7 mm margins in all directions. PBT planning was performed using 2–4 (median, 3) coplanar or non-coplanar beams of 230 MeV protons (Proteus 235; Ion Beam Applications, S.A., Louvain-la-Neuve, Belgium) to cover the PTV. The beam energy and spread-out Bragg peak, which was defined as the distance between the distal and proximal 90% points of the maximum dose value, were fine-tuned so that the at least 95% of the PTV was encompassed by 100% of the prescribed dose, and the proximal, distal, border smoothing, smearing and aperture margins for proton beams using the double scattering mode to PTV were set to 5–7 mm each. The beam weights of the plan were optimised to minimise the maximal dose within the target volume and OARs. The dose was calculated for the target volume and OARs with heterogeneity corrections and expressed in Gray equivalents [GyE = proton physical dose (in Gray) × relative biologic effectiveness (1.1)]. The treatment was designed so that at least 95% of the PTV would receive 100% of each prescribed dose, and the equivalent dose in 2-Gy fractions (EQD2, GyE_10_ or GyE_3_) was calculated using a linear quadratic model with α/β ratios of 10 and 3 for acute and late effects on tumour and OARs, respectively. The prescribed dose of PTV was 66 GyE (EQD2, 91.3 GyE_10_) in 10 fractions based on results of our previous [6, 7] and other studies [29, 33]. The details of the normal tissue constraints have been described previously [6, 31]: the maximum dose to the spinal cord could not exceed 27 GyE; the relative volumes of the total and remaining normal liver that received doses of 27 GyE (_TL_V27 and _RNL_V27) were below 60% and 50%, respectively; the absolute volumes of the oesophagus and stomach that received at least 37 GyE were ≤2 cm^3^; and the absolute volumes of the small and large bowel that received at least 35 GyE were ≤2 cm^3^. At each treatment fraction, digital orthogonal fluoroscopy was used to position patient and to verify the isocenter, and irradiation was performed during the exhalation phase using RPM system.

### Follow-up and statistical considerations

Patients were assessed weekly during hypofractionated PBT and after completion of PBT at 1 month followed by every 2 to 3 months for the first 2 years and every 6 months thereafter. Follow-up evaluations consisted of a physical examination, a complete blood count, liver-function testing, chest radiography, and liver dynamic contrast-enhanced CT or MRI. The responses of the primary tumour were defined as the maximal tumour response observed during the follow-up period unless progression occurred as determined by comparing CT and/or MRI scans before and after PBT using the modified Response Evaluation Criteria in Solid Tumors criteria (mRECIST) [34]. A complete disappearance of any intratumoural arterial enhancement in all target lesions was defined as a complete response (CR), a greater than 30% decrease of viable (enhancing) target lesion was defined as a partial response (PR), and a greater than 20% increase of viable target lesion within in-field target volume or death before evaluation of response after treatment was defined as progressive disease (PD). Patients who did not meet the criteria for PR or PD were categorised as having stable disease (SD). Acute haematological and non-haematological toxicities occurring within 3 months of PBT in the absence of disease progression were evaluated using Common Terminology Criteria for Adverse Events software version 3.0 (CTCAE v3.0).

Recurrence was proven pathologically by surgical resection, biopsy, or cytology and/or radiological findings showing an increase in size over time. Local progression was defined as a regrowth or new tumour within the treated volume. Intrahepatic recurrence was defined as a regrowth or new intrahepatic tumour outside the target volume. Distant metastasis was defined as lymph node recurrence, peritoneal seeding, or metastasis to extra-abdominal sites. Local progression-free survival (LPFS), relapse-free survival (RFS), and overall survival (OS) were defined as the intervals from the date of the start of PBT to the date of detection of local progression, any detection of recurrence, and death or last follow-up, respectively. OS rates were calculated using the Kaplan-Meier method. Univariate analysis of parameters predicting LPFS, RFS, and OS were assessed with log rank tests followed by multivariate analysis using Cox’s proportional hazard model with a forward selection procedure containing factors of *p* < 0.1 in univariate analysis. All statistical analyses were two-sided and were performed using STATA software (version 14.0; Stata Corp., College Station, TX, USA). A *p*-value < 0.05 indicated statistical significance.

## SUPPLEMENTARY MATERIALS FIGURE AND TABLE


