# Supplementary material for: Optimal time of tumour response evaluation and effectiveness of hypofractionated proton beam therapy for inoperable or recurrent hepatocellular carcinoma
Source: Oncotarget. 2017 Dec 19;9(3):4034–43. doi: 10.18632/oncotarget.23428 (PMC5790519; doi:10.18632/oncotarget.23428)
Supplement: Supplementary file 1 [file oncotarget-09-4034-s001.pdf]

Optimal time of tumour response evaluation and effectiveness of hypofractionated proton beam therapy for inoperable or recurrent hepatocellular carcinoma

SUPPLEMENTARY MATERIALS

Supplementary Table 1: Comparison of clinical characteristics according to tumour response

Characteristics		Tumour response		
		CR, *n* (%)	Non-CR, *n* (%)	*p*-value*
Gender	Male	55 (91.7)	5 (8.3)	1.000
	Female	11 (100)	0 (0)	
Age, years	<60	20 (90.9)	2 (9.1)	0.642
	≥60	46 (93.9)	3 (6.1)	
Aetiology of LC	HBV	53 (93.0)	4 (7.0)	1.000
	Others	13 (92.9)	1 (7.1)	
Child–Pugh Classification	A	64 (94.1)	4 (5.9)	0.199
	B	2 (66.7)	1 (33.3)	
AFP, ng/mL	<10	41 (95.3)	2 (4.7)	0.376
	≥10	25 (89.3)	3 (10.7)	
Tumour size, cm	<3	59 (92.2)	5 (7.8)	1.000
	≥3	7 (100)	0 (0)	
AJCC stage	I–II	61 (92.4)	5 (7.6)	1.000
	IIIA	5 (100)	0 (0)	
BCLC stage	A	41 (95.3)	2 (4.7)	0.367
	B	25 (89.3)	3 (10.7)	
Pre-Tx to PBT site	No	16 (100)	0 (100)	0.581
	Yes	50 (90.9)	5 (9.1)	
Pre-Tx to other site	No	10 (90.9)	1 (9.1)	0.581
	Yes	56 (6.7)	4 (9.3)	

Abbreviations: same as in Table 1.
*Fisher's exact test, two-tail.

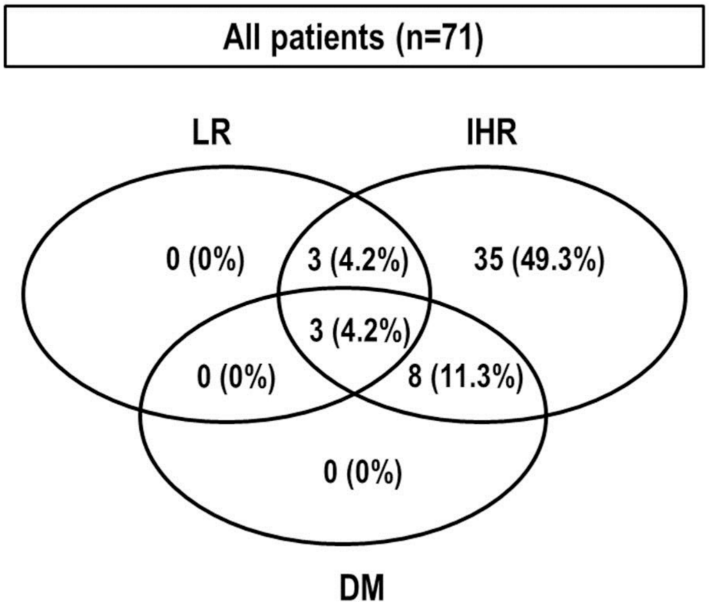

Supplementary Figure 1: Patterns of treatment failure in all patients. Abbreviations: LR, local recurrence; IHR, intrahepatic recurrence; and DM, distant metastasis.
